# The Association Between Postpartum Depression and Pica During Pregnancy

**DOI:** 10.5539/gjhs.v8n4p120

**Published:** 2015-07-30

**Authors:** Neda Ezzeddin, Roza Zavoshy, Mostafa Noroozi, Mohammad Ebrahim Sarichloo, Hassan Jahanihashemi

**Affiliations:** 1 Department of Nutrition, Faculty of Health, Qazvin University of Medical Sciences, Qazvin, Iran; 2 Children Growth Research Center, Qazvin University of Medical Sciences, Qazvin, Iran; 3 University of Social Welfare and Rehabilitation, Tehran, Iran

**Keywords:** Postpartum depression, pica, pregnancy

## Abstract

**Introduction and Objectives:**

Postpartum depression (PPD) is a common disorder and social debilitating that has adverse effects on the mother, child and family. Pica is an eating disorder characterized by persistent ingestion of substances that the consumer does not define as food. The aim of this study was to investigate the association of postpartum depression with pica during pregnancy.

**Method:**

This is case-control study was carried out in health centers in west Tehran. 152 depressed women (case group) and 148 non-depressed women (control group) were selected randomly from these health care centers. In addition to collecting demographic and pica data, the Edinburgh Depression Scale was used. The data was analyzed by both descriptive and analytic analyses such as chi-squared and logistic regression in SPSS version 16.

**Result:**

In this study, there wasn’t a significant association between PPD and pica during pregnancy (P=0.153, OR=2.043, CI=0.767, 5.438), but, postpartum depression has a significant association with type (clay) (P= 0.024) and duration (more than 2 months) (P= 0.023) of pica practice.

**Conclusions:**

In the present study, pregnancy pica was not important risk factor for PPD but there were similar risk factors such as iron supplementation during and postpartum pregnancy with pica and PPD.

## 1. Introduction

Postpartum depression (PPD) is a common and socially debilitating disorder, with adverse effects on mother, infant and family (Field, 2010). PPD is characterized by a depressed mood, excessive anxiety, insomnia and change in weight. The onset is generally within 12 weeks after delivery (Sadock & Sadock, 2008). Its etiology remains unknown, but risk factors affecting PPD includes: genetic predisposition, environmental, social, psychological and biological factors (Leung & Kaplan, 2009).

Eating disorders such as anorexia nervosa and bulimia have potentially adverse consequences on pregnancy including increased miscarriage, low birth weight, labor complications, and PPD. Symptoms of pregnancy and PPD are more prevalent among women with a history of eating disorders, and both anorexia nervosa and bulimia are associated with PPD (Leddy, Jones, Morgan, & Schulkin, 2009; Mazzeo et al., 2006).

Pica is an eating disorder characterized by continuous ingestion of substances that the eater does not consider as food including: earth, soil or clay (Geophagia), corn or laundry starch (Amylophagia) and ice or freezer frost (Pagophagia) (Mensah, Twumasi, Amenawonyo, Larbie, & Jnr, 2010; Young et al., 2010).

Pica is usually seen among pregnant and lactating women, children, the mentally lagged, and those with mental problems. Pica is not limited to a geographical area, race, sex, culture, or social position (Mortazavi & Mohammadi, 2010), and its prevalence during pregnancy varies from 0.02% to 74% (Boatin et al., 2012). The prevalence of pica in pregnant women has been reported 15.5% in Zahedan (a city in the southeastern of Iran) (Mortazavi & Mohammadi, 2010) and 22.3% in Kermanshah (a city in the West of Iran) (Rezavand & Rawshani, 2006). Etiology of pica is unknown, but some hypotheses attribute pica to deficiency of nutrients such as iron, calcium, copper, zinc, etc., leading to consumption of inedible substances containing these nutrients (Janbabai et al., 2011).

Many people with pica are ashamed to express it, as they consider it a mental disorder. Yet, it poses harmful consequences such as intestinal obstruction and perforation, preterm labor, mercury and lead poisoning, hyper- and hypokalemia, obesity, nutritional deficiency in pregnancy, anemia, iron deficiency, etc (Grotegut et al., 2006; Thihalolipavan, Candalla, & Ehrlich, 2013).

Previous studies have demonstrated the relationship of eating disorders such as anorexia nervosa and bulimia with PPD. However, few studies have investigated the relationship between PPD and pica. Since etiologies of pica and PPD are unknown, this study aims to determine the relationship between PPD and pregnancy pica.

## 2. Methods

### 2.1 Study Population, Design and Data Collection

This case-control cross-sectional study was carried out from March to May 2014, among women who had referred to health care centers supported by Shahid-Beheshti University of Medical Science in the west of Tehran. Eight urban health care centers were selected by random sampling among 16 centers. These 8 centers provided a good distribution of all 16 centers based on covered population and geographic area.

152 depressed women (case group) and 148 non-depressed women (control group) were selected randomly from these health care centers. Sampling technique was stratified (each center as stratum), number of samples for each center was proportional to the size of it.

The inclusion criteria were considered being 18-45 years old; 3-8 months elapsed from their delivery, with no history of depression and chronic disease (such as cardiovascular disease, diabetes, cancers and endocrine disease) and consent to participate in the study.

A two-part questionnaire was used, containing demographic details such as age, education, occupation, infant's gender, pregnancy ranks, history of abortion or stillbirth, and also pregnancy pica.

The questionnaire of pica during pregnancy included: the kind (ice, freezer frost, soil, rosary and praying clay, raw starch, cigarette ashes, oil or petrol, baking soda, tobacco, charcoal and or any other non-food substances that eaten during pregnancy and not listed above), the onset, duration, frequency (daily, weekly or monthly) and the reasons (unable to give them, satisfy craving, relief of nausea, reduced stress and anxiety and or other reasons) of pica practice.

### 2.2 Measures

#### 2.2.1 Assessment of Depression

To identify women with PPD, Edinburgh Postpartum Depression Scale (EPDS) was used, which contains 10 items on the person's feelings over the past 7 days, each with 4 options, scoring from 0 to 3, with maximum score of 30 and minimum of zero. In this study, women scoring above 10 were considered depressed, and those scoring 10 and below, non-depressed. This is a standard and approved questionnaire with reliability of 0.9 and in Persian language (Alipour, Lamyian, & Hajizadeh, 2012).

#### 2.2.2 Anthropometric Assessment

Mothers’ height was measured in standing position, with no shoes, heels touching the wall, and face looking forward using Seca stadiometer with the precision of 0.1 cm. Mothers’ weight was measured wearing minimum clothing and no shoes using Seca digital weighing scales. BMI was calculated by dividing weight (kg) over height squared (m^2^).

### 2.3 Statistical Analysis

The data were analyzed using statistical tests, including chi-squared, logistic regression, using the Statistical Package for Social Sciences version 16 (SPSS Inc., Chicago, IL, USA).

## 3. Results

Mean age was 28.61±6.07 years in depressed group and 28.82±5.22 years in non-depressed group. Case (152 women) and control (148 women) groups were matched in terms of age, BMI, infant's sex, pregnancy ranks, and history of abortion or stillbirth. The result of study showed no association between mother's occupation and PPD (P=0.441). The prevalence of under diploma women in depressed group was significantly higher than non-depressed group (P=0.033) (Table 1).

**Table 1:** The association between PPD and some demographics and obstetrics variables among studied mother

Variables	depressed	Non-depressed	P-value*
	(%)N	(%)N	
Mother age			
*<26*	48(52.2)	44(47.8)	
*26-34*	81(50)	81(50)	0.941
*35>*	23(50)	23(50)	
Mother BMI			
*< 18.5*	6(75)	2(25)	
*18.5-24.9*	68(48.6)	72(51.4)	0.288
*25-29.9*	50(52.6)	45(47.4)	
*> 30*	14(40)	21(60)	
pregnancy ranks			
*1*	72(47.4)	72(48.6)	
*2*	50(32.9)	47(31.8)	0.972
*>2*	30(19.7)	29(19.6)	
type of delivery			
*NVD*	30 (43.5)	39(56.5)	
*Caesarean*	122(52.8)	109(47.2)	0.173
the history of abortions or stillbirths			
*Yes*	34(22.4)	32(21.6)	
*No*	118(77.6)	116(78.4)	0.876
infant sex			
*Female*	75(48.7)	79(51.3)	
*Male*	76(52.8)	68(47.2)	0.482
Mother occupational status			
*Employment*	134(51.5)	126(48.5)	
*Housekeepers*	18(45)	22(55)	0.441
Education level of mother			
*Under diploma*	40(62.5)	24(37.5)	
*Diploma and more*	112(47.5)	124(52.5)	0.033
Iron supplementation During pregnancy			
*Yes*	120(48.2) 32(62.7)	129(51.8)19(37.3)	0.058
*No*			
			
Iron supplementation after pregnancy			
*Yes*	53(37.3) 99(62.7)	89(62.7) 59(37.3)	< 0.001
*No*			

* Chi-square test.

The prevalence of pica was 11.8% in depressed women, and 4.7% in non-depressed women (P=0.026, OR=2.7), which meant that women with pregnancy pica were 2.7 times more likely to be affected by PPD (table 2). Sixty-four percent of women were reported practicing pica regularly on daily basis and Pagophagia (ice and freezer frost) was the most common non-food substance (76%, n=19) followed by Rosary and praying clay (24%, n=6). 64% of women experienced pica daily and Pica was most prevalent (84%, n=21) in the first trimester followed by 12% (n=3) and 4% (n=1) in the second and third trimester respectively. Ten women (40%) had no reason for pica, but 4 (16%) had severe craving, 1 (4%) wanted to reduce nausea, 8 (32%) wanted to reduce stress and anxiety, and 2 (8%) expressed other reasons. Women who practiced long term pica during pregnancy, were more prone to PPD (P=0.023), and all women with rosary and praying clay picas (n=6) were affected by PPD (P=0.024) (table 3). The results of study showed a significant association between regular iron supplementation during pregnancy and pica practice (P< 0.001) (Table 4), also there were significant association between postpartum iron supplementation with PPD (P< 0.001). Iron supplementation during pregnancy also had protective effect against PPD but wasn’t statistically significant (p=0.058) (Table 1).

**Table 2:** The association between PPD and Pica during pregnancy

groups	Pica during pregnancy		Total	P-value*	OR** (95% CI***)

	No	Yes			
	N (%)	N (%)	N (%)		
Non-depressed	141(95.3)	7(4.7)	148(100)		
Depressed	134(88.2)	18(11.8)	152(100)	0.026	2.70 (1.095-6.685)
Total	275(91.7)	25(8.3)	300(100)		

* Chi-square test;

** OR = odds ratio;

*** CI =confidence interval.

**Table 3:** The association between Length and kind of pregnancy Pica with PPD

Variables	Length of Pica during pregnancy (month)			Type of pica		
	(%)N			(%)N		
	*0*	*≥2*	*> 2*	*Nothing*	*Ice and Freezer frost*	*Rosary and praying clay*
**Depressed**	134(48.7)	2(40)	16(80)	134(48.7)	12(63.2)	6(100)
**Non-depressed**	141(51.3)	3(60)	4(20)	141(51.3)	7(36.8)	**0**
**Total**	275(100)	5(100)	20(100)	275(100)	19(100)	6(100)
P-value^*^	0.023			0.024		

**Table 4:** The association between iron supplementation during pregnancy and pica practice

Variables	Pica practice			P-value*

	No	Yes	Total	
	N (%)	N (%)	N (%)
**Iron supplementation during pregnancy**				
*Yes*	237(95.2)	12(4.8)	249(100)	P< 0.001
*No*	38(74.5)	13(25.5)	51(100)	

* Chi-square test.

By adjusting the confounding factors affecting depression in logistic regression, only iron supplementation after pregnancy was significant (Table 5).

**Table 5:** Final logistic regression model for predicting PPD

variables	P-value	OR*	95 % C.I**	
			Lower	Upper
**Maternal education**	0.201	1.485	0.81	2.723
**Pica during pregnancy**	0.153	2.043	0.767	5.438
**Iron supplementation during pregnancy**	0.822	1.084	0.536	2.191
**Iron supplementation after pregnancy**	< 0.001	2.705	1.628	4.496

*Note.* Table is based on results of binary logistic regression (only for significant situations based on chi-square Test on Table 1).

* Odds ratio

** Confidence Interval.

## 4. Discussion

PPD is a common and multi factorial psychiatric disorder with biological, psychological, and sociological aspects interacting with mothers risk individually (Klainin & Arthur, 2009; Sooki et al., 2012). According to the various studies, women whit eating disorder such as anorexia nervosa and bulimia are exposed to different complications, including PPD (Leddy et al., 2009; Mazzeo et al., 2006; Meltzer-Brody et al., 2011). In this study, we assessed another form of eating disorder known as pica. The results of study showed that women who practiced pica during pregnancy, were more likely to PPD but by adjusting in logestic regression, it wasn’t statistically significant. There are very few studies on the association between pregnancy pica and PPD, and since the prevalence of pica is high in some African countries and Latin America (López & Langini, 2007; Nyaruhucha, 2009), more studies may be helpful.

Stress and anxiety is known as a risk factor for pica behavior in some studies. In case control study, an adult female patient ingested chalk in stressful situation (Bhatia & Gupta, 2009). Stressful life events also identified as an important predictors of PPD (Robertson, Grace, Wallington, & Stewart, 2004). We didn’t assess women Stress and anxiety during pregnancy but 32% (table 6) of women declared that pica reduced their stress and anxiety during pregnancy and all of them practiced PPD. We conclude that maybe Stress and anxiety during pregnancy is a cause of pica practice during pregnancy and PPD after delivery.

**Table 6:** Frequency of Pica reasons by women in depressed and non-depressed groups

groups	Pica reasons by women N (%)				

	Unable to say	satisfy craving	Relief of nausea	reduced stress and anxiety	Other reasons
**Non-depressed**	4 (40)	1(25)	1(100)	0(0)	1(50)
**depressed**	6(60)	3(75)	0	8(100)	1(50)
**Total**	10(100)	4(100)	1(100)	8(100)	2(100)

Anemia is known as important risk factors for pica behavior (Khoushabi et al., 2014; Rezavand & Rawshani, 2006; Saunders et al., 2009; Young et al., 2010). In some studies, there were also significant association between Anemia and PPD (Beard et al., 2005; Goshtasebi, Alizadeh, & Gandevani, 2013). In this study, pregnancy and postpartum anemia not assessed but the results of study showed a significant association between iron supplementation during pregnancy and pica practice. Iron supplementation during and postpartum also had a protective effect against PPD. In systematic Reviews, Iron supplements aimed at preventing PPD (Miller, Murray, Beckmann, Kent, & Macfarlane, 2013). A hypothesis about the association between PPD and pregnancy pica may related to anemia that manifest itself as a pica during pregnancy and PPD after delivery. So suggested that future studies assess anemia during and postpartum pregnancy when evaluate this association.

**Limitation and Conclusions**

In the present study, we tried to limit and match some confounding variables in assessing the relationship between PPD and pregnancy pica. So, recommended that future studies be conducted longitudinally and neutralization of other variables.

In this study, there wasn’t significant association between PPD and pica practice. In others studies, the association between another forms of eating disorders examined but pica practice not well studied. Given the importance of the subject on the individual and the community, further studies in this area are recommended.

In the present study, iron supplementation during and postpartum were an important factor against pica practice and PPD. Although iron supplementation for pregnant women is free in Iranian health care centers but some women don’t take iron supplements. Health practitioners should justify pregnant women about importance of taking supplements. In this study, pregnancy pica assessed 3-8 month after delivery so, maybe the prevalence of pica have been underestimated because of forgetfulness. Also, there are other variables may affect incidence of postpartum depression.
